# A retrospective study on the investigation of potential dosimetric benefits of online adaptive proton therapy for head and neck cancer

**DOI:** 10.1002/acm2.14308

**Published:** 2024-02-18

**Authors:** Chih‐Wei Chang, Duncan Bohannon, Zhen Tian, Yinan Wang, Mark W. Mcdonald, David S. Yu, Tian Liu, Jun Zhou, Xiaofeng Yang

**Affiliations:** ^1^ Department of Radiation Oncology and Winship Cancer Institute Emory University Atlanta Georgia USA; ^2^ Department of Radiation and Cellular Oncology University of Chicago Chicago Illinois USA; ^3^ Department of Radiation Oncology Mount Sinai Medical Center New York New York USA

**Keywords:** head‐and‐neck cancer, offline adaptive proton therapy, online adaptive proton therapy, treatment outcome

## Abstract

**Purpose:**

Proton therapy is sensitive to anatomical changes, often occurring in head‐and‐neck (HN) cancer patients. Although multiple studies have proposed online adaptive proton therapy (APT), there is still a concern in the radiotherapy community about the necessity of online APT. We have performed a retrospective study to investigate the potential dosimetric benefits of online APT for HN patients relative to the current offline APT.

**Methods:**

Our retrospective study has a patient cohort of 10 cases. To mimic online APT, we re‐evaluated the dose of the in‐use treatment plan on patients’ actual treatment anatomy captured by cone‐beam CT (CBCT) for each fraction and performed a templated‐based automatic replanning if needed, assuming that these were performed online before treatment delivery. Cumulative dose of the simulated online APT course was calculated and compared with that of the actual offline APT course and the designed plan dose of the initial treatment plan (referred to as nominal plan). The ProKnow scoring system was employed and adapted for our study to quantify the actual quality of both courses against our planning goals.

**Results:**

The average score of the nominal plans over the 10 cases is 41.0, while those of the actual offline APT course and our simulated online course is 25.8 and 37.5, respectively. Compared to the offline APT course, our online course improved dose quality for all cases, with the score improvement ranging from 0.4 to 26.9 and an average improvement of 11.7.

**Conclusion:**

The results of our retrospective study have demonstrated that online APT can better address anatomical changes for HN cancer patients than the current offline replanning practice. The advanced artificial intelligence based automatic replanning technology presents a promising avenue for extending potential benefits of online APT.

## 1 INTRODUCTION

Proton radiotherapy (RT) has the Bragg peak at well‐defined ranges and no exit doses, offering unique advantages in the sparing of critical organs, particularly for head and neck (HN) cancer that often involves multiple critical organs at the treatment site. It has been reported that compared with intensity‐modulated RT (IMRT) and volumetric modulated arc therapy (VMAT), intensity‐modulated proton therapy (IMPT) can reduce the mean doses to the larynx and parotid glands^1^ and hence improve the quality of life in HN cancer patients.^2^, ^3^

Radiation treatment plans are typically designed based on a computed tomography (CT) scan acquired a few weeks prior to the treatment course, and the treatment is delivered over 4−6 weeks. The HN anatomy is prone to considerable volumetric and positional change over this time frame due to resolving postoperative edema, tumor shrinkage, changes in overall body weight, and daily treatment setup variations. As the range of the Bragg peak highly depends on the tissue density along the beam path, proton RT is very sensitive to anatomical changes. The treatment plan, that is, designed based on the patient's original anatomy can result in mispositioned Bragg peaks in the patient's current anatomy, leading to the underdosing of tumors and/or overdosing of the critical organs. Although robust plan optimization^4^ can account for the anatomical changes to some extent, this method can maintain the designed plan quality only when the bounding scenarios cover the patient's actual treatment anatomy during the robust optimization.^5^, ^6^ Adaptive proton therapy (APT) offers a general solution for patients’ anatomical changes without increasing the dose to critical organs and normal tissues.^7^ Many proton centers have started to acquire weekly or bi‐weekly quality assurance CT (QACT) images to regularly monitor the anatomical changes of HN cancer patients throughout the treatment course, and on‐demand offline replanning can be performed if necessary. Unlike non‐adaptive IMPT, offline adaptive IMPT can significantly reduce the mean doses to the larynx and parotid gland.^1^ However, due to the intensive work required by the current APT technology, it typically takes about a week from the QACT acquisition to the time the new treatment plan is ready for review. The old plan often has to be continually used until the new plan is ready due to the danger of accelerated tumor repopulation caused by breaks in the treatment course.

The online APT can potentially be a desired solution, in which the actual plan quality of the original plan on the patient's up‐to‐date anatomy after treatment setup is assessed at each fraction, and on‐demand rapid replanning can be performed for the current fraction while the patient is lying on the treatment couch. However, it is challenging and still unavailable in proton clinics due to the lack of a high‐quality onboard 3D imaging system and the long time required for a complex and labor‐intensive workflow.^8^ Although multiple groups^9^, ^10^, ^11^, ^12^ have been endeavoring to overcome these challenges to enable online APT, there is also a concern in the RT community about the necessity of online APT. For instance, it is possible that the dosimetric disadvantages of the current offline APT shown in specific treatment fractions might be smeared due to the daily setup variations and hence counteracted in the cumulative dose over the entire treatment course, which might dampen the need for devoting significant efforts to develop the online APT technology.

This study aims to investigate the potential dosimetric benefits of online APT for patients with anatomical changes during treatment and compare it with the current offline APT practice. Online APT technology is currently unavailable, so we have performed a retrospective simulation study with a cohort of 10 HN cancer patients who required manual offline replanning on a repeated CT scan during their actual treatment courses. In this study, we have recalculated and evaluated the treatment plan's actual dose on the patient's treatment anatomy captured by cone beam CT (CBCT) at each fraction. The cumulative dose of the simulated “online” APT course was compared with the cumulative dose of the offline APT course that was used for the actual patient treatment, as well as with the initially designed plan dose of the original treatment plan generated prior to the treatment course (referred to as the nominal plan). We hope that this simulation study might shed some light on the potential benefit of online APT to help the RT community and vendors determine whether it is worth devoting significant efforts to implement online APT.

## 2 MATERIALS AND METHODS

### 2.1 Patient data and the current offline APT workflow

We have retrieved clinical data of 10 HN cancer patients, who were treated with IMPT at our institution and had at least one offline replanning during their treatment courses, from our institutional database for the retrospective study. Table 1 summarizes the actual offline APT treatment information for the 10 HN patients. In their actual offline APT treatment, the CT images acquired prior to the start of the treatment course and used for the nominal treatment planning (referred to as planning CT) were acquired using a Siemens SOMATOM Definition Edge scanner. The treatment planning system RayStation 9A (RaySearch Lab., Stockholm, Sweden), was used to perform the nominal treatment planning following our institutional planning guidelines. Our RayStation is running on a clinical server with dual Intel Xeon Gold 6136 CPU (2017 model year), 512GB RAM, and an NVIDIA Quadro RTX 8000. It supports GPU‐based Monte Carlo dose calculation and deformable image registration. All the clinical plans were generated using robust plan optimization, with 3.0 mm setup positional uncertainty in the three orthogonal directions and 3.5% range uncertainty. With the combination of 3 range uncertainty levels (± 3.5% and 0%) and 7 positional uncertainties (anterior/posterior, left/right, superior/ inferior, and no positional uncertainty), a total of 21 scenarios were considered in robust plan optimization.

**TABLE 1:** Summary of the information of the actual offline APT treatment courses of the 10 HN patients.

		Patient 1	Patient 2	Patient 3	Patient 4	Patient 5	Patient 6	Patient 7	Patient 8	Patient 9	Patient 10
CTV‐High (cm^3^)	Initial volume	60.36	84.40	68.82	57.67	107.90	10.24	96.62	304.31	26.15	235.21
	Final volume	61.87	78.73	58.46	59.04	98.06	9.90	82.96	247.87	25.61	204.53
CTV‐Mid (cm^3^)	Initial volume	145.59	122.69	132.89	84.46	–	207.09	140.47	–	–	–
	Final volume	144.37	115.39	136.69	81.94	–	200.16	114.67	–	–	–
CTV‐Low, (cm^3^)	Initial volume	404.53	219.41	180.18	270.44	240.13	263.44	471.0	–	263.38	516.64
	Final volume	405.59	189.75	160.75	243.47	223.78	260.07	444.78	–	222.79	443.40
Prescription dose (Gy‐RBE)	CTV‐High	68.4	70	70	70	70	66	70	60	70	60
	CTV‐Mid	64.8	63	60.2	63	–	60.06	60.2	–	–	–
	CTV‐Low	54	56	53.9	56	53.9	54.12	53.9	–	53.9	54
Number of fractions		36	35	35	35	35	33	35	30	35	30
Replan times		1	2	1	1	1	1	1	2	2	2
Replan reasons		Anatomy change	CTV coverage loss	Anatomy change	Anatomy change, OAR overdose	Hot spot	Hot spot	Hot spot, OAR overdose	Anatomy change, hot spot, CTV coverage loss	CTV coverage loss	Anatomy change

Abbreviations: CTV‐High, the high‐risk clinical target volume; CTV‐Mid, intermediate‐risk clinical target volume; CTV‐Low, the low‐risk clinical target volume, respectively.

At our institution, most HN IMPT plans were optimized using five proton beams, including left posterior oblique (LPO), left anterior oblique (LAO), anteroposterior (AP), right anterior oblique (RAO), and right posterior oblique (RPO). A constant relative biological effectiveness (RBE) of 1.1 was assumed for proton radiation doses based on IAEA/ICRU guidelines.^13^, ^14^ The prescribed doses ranged from 68.4 to 70 Gy‐RBE with 1.9–2.0 Gy‐RBE daily fractions. CBCT images were acquired at the beginning of each treatment fraction to guide the treatment setup. During the treatment course, bi‐weekly QACT images were acquired on the same CT scanner to regularly monitor the patient's anatomical change and assess the actual plan quality of the nominal plan on the new anatomy captured by QACT. A full offline replanning was triggered, either when the obtained dose volume metrics of the recalculated nominal plan did not satisfy our institutional criteria any longer or when a substantial anatomical change was observed on a treatment CBCT image set. For the offline replanning, a deformable registration between the planning CT images and the most recent QACT images was first performed to propagate the contours of clinical target volumes (CTVs) and organs at risk (OARs) from the planning CT onto the QACT. After the attending physician reviewed the propagated contours and modified the contours if needed, replanning was then performed by one of our proton dosimetrists using RayStation following the same planning guideline used in the nominal treatment planning. Table 2 lists our institutional planning guideline that was used for these patients.

**TABLE 2:** Our institutional planning guideline used for the 10 HN patients.

Structure	Planning goals
CTV‐High	At least 98% of its volume receives ≥100% of its prescription dose
	Maximum dose is no more than 107% of its prescription dose
CTV‐Mid	At least 98% of its volume receives ≥100% of its prescription dose
CTV‐Low	At least 98% of its volume receives ≥100% of its prescription dose
Right Parotid	Mean dose is no more than 26 Gy‐RBE
Left Parotid	Mean dose is no more than 26 Gy‐RBE
Oral cavity	Mean dose is no more than 35 Gy‐RBE
Brainstem	No more than 0.03 cm^3^ of its volume receives a dose higher than 63 Gy‐RBE
Spinal cord	No more than 0.03 cm^3^ of its volume receives a dose higher than 54 Gy‐RBE

### 2.2 Our proposed workflow of CBCT‐guided online APT

**FIGURE 1:**
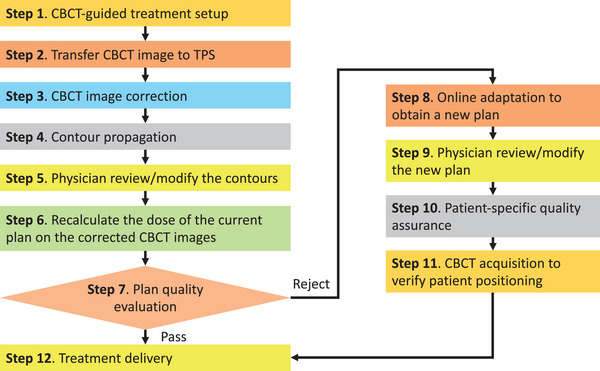
The proposed workflow of CBCT‐guided online APT used in this retrospective study.

Figure 1 depicts our proposed workflow of the CBCT‐guided online APT for the existing proton systems, which consists of the following steps:

*Step 1:* At the beginning of each treatment fraction, the CBCT images will be acquired and registered to the planning CT images via rigid image registration to determine the couch movement needed to reproduce the planned treatment position, which is the same as the current offline APT.
*Step 2 :* The CBCT images and the subsequent couch movement values will then be retrieved from ARIA oncology information system (V16.1, Varian Co., Palo Alto, CA, USA) through an internal network and imported into RayStation. Note that the couch movement will be applied to the retrieved CBCT images to ensure that the actual treatment setup with the patient's actual anatomy will be used to evaluate the plan quality of the current in‐use treatment plan.
*Step 3 :* The acquired CBCT images cannot be directly used for daily dose evaluation and online plan adaptation, as the existing on‐board CBCT imaging system suffers from severe image artifacts mainly due to scatter contamination, which impairs the soft tissue contrast and leads to large Hounsfield Unit (HU) uncertainties. Hence, we will perform CBCT image correction at this step using our previously developed CBCT correction framework^15^ Specifically, we will deform the planning CT images to the daily CBCT images via deformable image registration, using the ANACONDA algorithm^16^ provided in RayStation, in order to use the correct HU numbers from the planning CT images while preserving the patient's actual anatomy captured on the CBCT images. In addition, as the nasal cavity filling can vary from time to time, we will copy the air cavities from the CBCT images to the deformed planning CT images to preserve the actual nasal cavity filling. The resulting final images are referred to as corrected CBCT images in the rest of this manuscript.
*Step 4 :* We will propagate the contours of the CTVs and OARs from the planning CT images to the corrected CBCT images using RayStation, similar to the contour propagation from the planning CT images to the QACT images in the current offline APT practice. Note that users have the flexibility to specify the propagation of each contour to be either a rigid copy or a deformable mapping in RayStation.
*Step 5 :* Attending physician will review the propagated contours and modify the contours if needed (the final contours are referred to as daily contours in the rest of this manuscript).
*Step 6 :* We will recalculate the current in‐use treatment plan dose on the corrected daily CBCT images.
*Step 7 :* We will calculate the dose volume histograms (DVHs) for each CTV and OAR using the daily contours to evaluate the actual plan quality. If the actual plan quality does not satisfy our clinical goals or is substantially worse than the nominal plan, online adaptation will be triggered to conduct steps 8−11.
*Step 8 :* If online adaptation is triggered, a treatment plan optimization will be performed using the corrected CBCT images and the daily contours to generate a new plan that will satisfy the clinical goals.
*Step 9 :* Attending physician will review the plan and modify the plan if needed.
*Step 10 :* The approved new plan will be exported to the treatment delivery system. An independent secondary dose calculation will be performed on the exported new plan using the corrected CBCT images, which will serve as a patient‐specific pre‐delivery quality assurance for online APT to ensure patient safety.
*Step 11 :* Another CBCT scan will be acquired before treatment delivery of the new plan to ensure the same treatment positioning as on the corrected CBCT images, in case that the patient might move while waiting for the new plan.
*Step 12 :* Treatment delivery. After treatment delivery of the new plan, the machine delivery log file, which records the actual machine parameters during treatment delivery at high temporal frequency, will be retrieved. The specific information of the delivered beam spots will be extracted from the log file to perform another dose calculation using the corrected CBCT images, as a patient‐specific post‐delivery quality assurance for online APT.^17^, ^18^, ^19^, ^20^, ^21^, ^22^

### 2.3 Retrospective simulation study

As mentioned in the introduction session, our purpose of this retrospective study is to investigate the potential dosimetric effect of online APT, if successfully developed in the future, for HN cancer patients with observed anatomical changes during the treatment course, and compare it with the current offline APT practice. Because online APT is not available in proton clinics yet, in our study we have imported the daily CBCT images, that were acquired from each treatment fraction of the actual offline APT treatment of the selected 10 HN patients, into RayStation to conduct steps 3−10 of the proposed workflow to simulate an online APT treatment course. In‐house Python scripts have been developed to streamline the workflow in the RayStation.

In our simulation study, when online plan adaptation was triggered, a new plan optimization was performed using the corresponding corrected CBCT images and the daily contours. Specifically, the same beam arrangement that was used in the nominal treatment planning of the same patient was used in our online replanning. Considering the stringent time requirement of online APT, we did not perform a full plan optimization, which often involves a trial‐of‐error process of manual adjustment of the planning constraints and their relative priorities, as we do for the nominal treatment planning or the offline replanning. Instead, in our simulation we employed the template‐based automatic replanning strategy^23^, ^24^ used by Ethos (Varian Co., Palo Alto, CA, USA), a CBCT‐based online adaptative photon therapy system. This template‐based replanning strategy adopts the same planning constraints and priorities, which were specified and used to generate the nominal plan, for online adaptation. In addition, unlike the nominal treatment planning or the offline replanning, we did not use 21 scenarios in the robust plan optimization for our simulated online adaptation, as the actual treatment setup at that specific treatment fraction was already captured in the corrected CBCT images. Instead, only three scenarios of different range uncertainties (i.e., 0% and ±3.5%) were included in the robust plan optimization, which significantly reduced the time taken by online replanning. Meanwhile, a 1.5 mm margin was added to the CTV volumes to account for the potential intrafraction motion and the spot position uncertainty. The 1.5 mm margin size was determined based on two study findings. Firstly, an investigation on the intra‐fractional motion of HN cancer patients with the aid of an immobilization device (e.g., Orfit 5‐point mask with shoulder extension and headrest cushion) reported a motion range of within 1 mm translation and 0.7° rotation.^25^ Secondly, an assessment study on the spot position uncertainty of scanning proton pencil beam reported an uncertainty range of within 1 mm for the maximum day‐to‐day variance for any given spot positions.^26^

After simulating the CBCT‐guided online APT for every treatment fraction by conducting the steps 3−10 of the proposed workflow, deformable image registration was performed to deform the corrected CBCT images to the planning CT images to accumulate the actual dose of the in‐use plan (i.e., either the nominal plan if online adaptation was not triggered yet or the latest new plan) over the entire treatment course. For comparison purposes, the actual dose of the in‐use plan of each treatment fraction of the actual offline APT treatment course was also recalculated with the corresponding corrected CBCT images and accumulated over the entire offline APT course similarly. In our study, we compared the cumulative dose distributions of the simulated “online” APT treatment course and the actual offline APT treatment course for the ten HN cases, with respect to the originally planned dose of the nominal treatment plan, to investigate the potential dosimetric effect of online APT.

**FIGURE 2:**
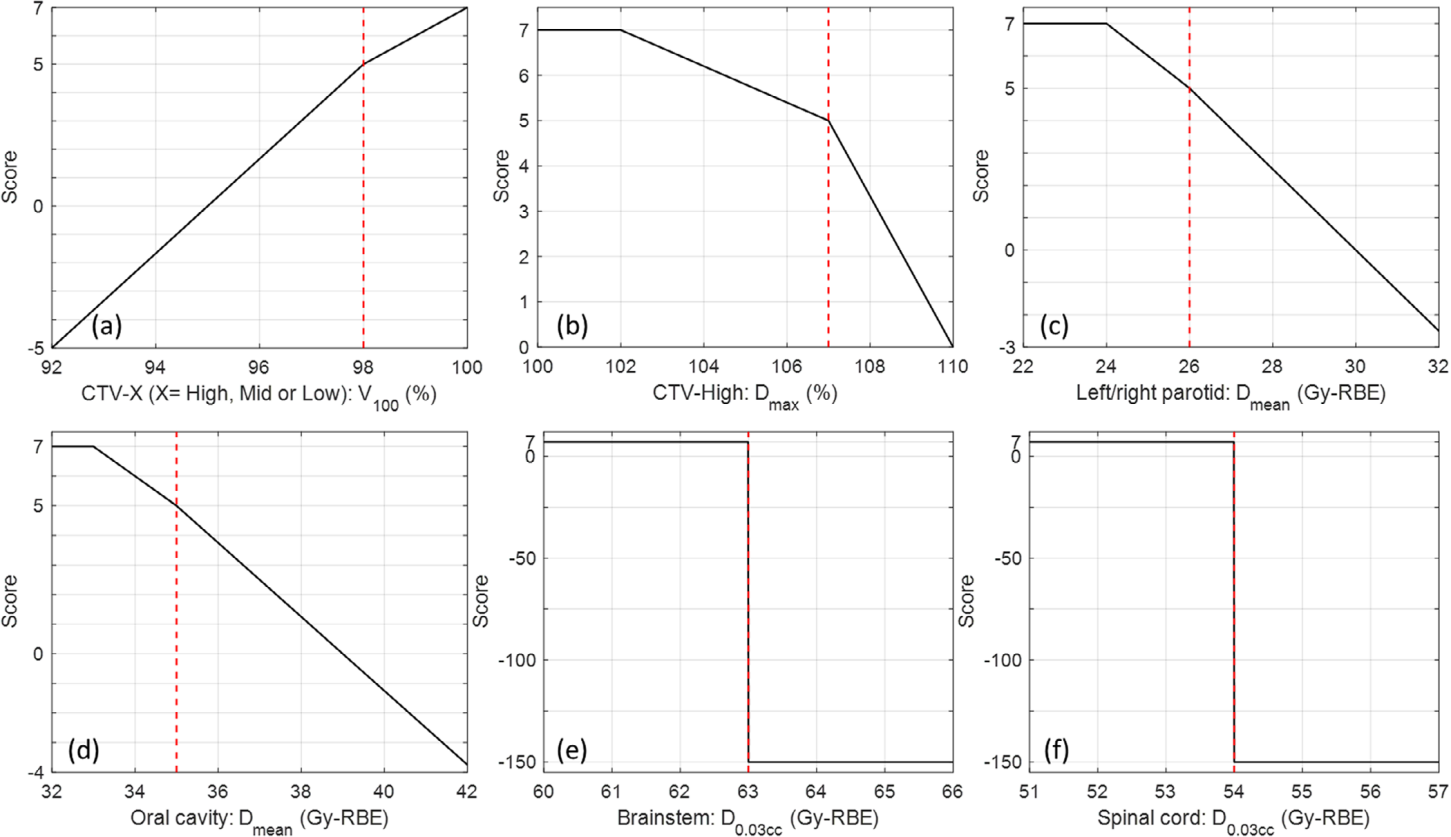
The modified ProKnow scoring functions that were used in this retrospective study to quantify the quality of the cumulative dose of the in‐use treatment plans on patient's actual treatment anatomy. *V*_100_ denotes the CTV percentage volume that received at least 100% of the prescription does. *D*_max_ denotes the maximum dose received by CTV‐High and is present in the percentage of prescription dose. *D*_mean_ denote the mean dose received by an OAR. *D*_0.03cc_ denotes the minimal dose received by the highest irradiated volume of 0.03 cm^3^ of an OAR. The maximum score of each scoring metric is 7. The red dash lines represent our institutional clinical planning goals (as listed in Table 2).

To ease this dose comparison, we employed a scoring system of ProKnow (ProKnow Systems, Elekta, Sanford, FL, USA) to quantify the actual quality of the offline APT course and the simulated online APT course against our clinical planning goals for the 10 patients. The ProKnow scoring system was originally designed to quantify the plan quality of HN IMRT plans for treatment planning competitions, and has been employed by multiple studies on automated planning for algorithm development and quantitative evaluation of the resulting plan quality.^27^, ^28^, ^29^ Note that we did not include all the scoring criteria from the original ProKnow scoring system, since we only consider those that were involved in our planning guidelines for the 10 IMPT patient cases. Specifically, the scoring system used in this study is the equally‐weighted sum over multiple scoring functions, each corresponding to a clinical criterion of our institutional planning guideline, including CTV dose coverage, hot spot inside the high‐risk CTV (denoted as CTV‐High), mean doses of left parotid and left parotid, mean dose of the oral cavity, and the minimal doses received by the highest irradiated 0.03 cm^3^ volume (denoted as *D*_0.03cc_) of brainstem and spinal cord. Three modifications were made to the original ProKnow scoring functions to make them applicable to our study. Firstly, given that our institutional clinical goals for CTV dose coverage and CTV‐High hot spot for HN IMPT plans are set to be ≥98% and ≤107%, respectively, which are different from the clinical goals specified in the original ProKnow scoring system for HN IMRT plans (i.e., ≥95% and ≤105%), we shifted the two corresponding scoring functions to align with our specific clinical goals. Secondly, regarding the scoring functions for sparing the brainstem and spinal cord, which are serial organs, the ProKnow scoring system assigns a negative score of −150 if the *D*_0.03cc_ dose exceeds the specified tolerance. This signifies that it is typically considered clinically unacceptable for the *D*_0.03cc_ dose of the brainstem or spinal cord to exceed the specified tolerance. On the other hand, a full score of 0 is assigned as long as the *D*_0.03cc_ dose remains within the tolerance, which is based on the consideration that their highest irradiated small volume for successive radiotherapy courses typically does not coincide at the same location. In this study, to be consistent with other scoring functions whose full score values are all set to be 7, we set the full scores of the brainstem and spinal cord sparing to be 7 as well. Thirdly, all the Proknow scoring functions (excluding the two for the brainstem and spinal cord) have a range from 0 to 7, without distinguishing between plans whose endpoints exceed the tolerance beyond a certain extent. For instance, ProKnow assigns a score of 0 to all plans with a mean dose of the right parotid higher than 30 Gy. Recognizing that a patient's anatomical changes during the treatment course may significantly deteriorate the quality of the offline and online APT courses compared to the nominal plan quality, we modified the original ProKnow scoring functions to allow for negative scores, which enables us to further quantify the quality of inferior plans. Our adapted scoring functions for this study are illustrated in Figure 2, where the red dashed lines correspond to the clinical goals outlined in our institutional planning guidelines.

## 3 RESULTS

In our retrospective study, it took about 2 min to carry out steps 1−7 of the proposed online adaptation workflow for each patient. For step 8 of the online replanning, with the template‐based replanning strategy, which employed the same priorities that were used in the original treatment planning for fast online adaptation without any manual fine‐tuning, it took about 10−15 min for each patient. Table 3 presents the CTV and OAR dose–volume endpoints of the 10 patient cases obtained by the planned dose of the nominal plan, the cumulative dose of the actual offline APT treatment course, and the cumulative dose of the simulated online APT course, respectively. For each dose, its quality score corresponds to each planning criterion of our institutional planning guideline, and the total scores are presented in Table 4. Score comparison results among the three dose distributions for each patient are presented in Table 5. The total scores of the nominal plans for the 10 cases range from 4.0 to 57.1, with an average score of 41.0. We would like to emphasize that the optimal plan quality achievable for each patient is fundamentally influenced by their unique anatomy. The score values of the nominal plans for the 10 cases also serve as indicators of the planning difficulty level for each patient to some extent, considering that these nominal plans were approved for clinical treatment and should be of high quality. Compared to the nominal plans, the average score for the offline APT course over the 10 cases is 25.8, and the average score for our simulated online APT course is 37.5. Compared to the offline APT course, our online APT course improved the total score for all 10 cases, with the enhancement ranging from 0.4 to 26.9 and an average improvement of 11.7, which demonstrates that online adaptation generally can better address patients’ anatomical changes than the current offline replanning practice.

**TABLE 3:** Dose volume endpoints of CTVs and OARs for the 10 patients, obtained by the nominal plan, the cumulative dose of the actual offline APT course, and the cumulative dose of the simulated online APT course, respectively.

		CTV‐High			CTV‐Mid		CTV‐Low		Right Parotid	Left Parotid	Oral Cavity	Brainstem	Spinal Cord
		*V*_100_ (%)	*D*_98_ (%)	*D*_max_ (%)	*V*_100_ (%)	*D*_98_ (%)	*V*_100_ (%)	*D*_98_ (%)	*D*_mean_ (Gy‐RBE)	*D*_mean_ (Gy‐RBE)	*D*_mean_ (Gy‐RBE)	*D*_0.03cc_ (Gy‐RBE)	*D*_0.03cc_ (Gy‐RBE)
Patient 1	Nominal	90.4	87.6	106.4	96.3	94.5	99.1	101.3	2.4	18.9	6.6	62.9	10.6
	Offline	83.3	86.4	107.8	94.8	93.8	97.9	99.9	1.6	19.6	6.5	61.2	9.2
	Online	84.2	88.9	103.8	96.3	96.6	99.2	101.5	2.1	18.4	6.4	63.0	11.3
Patient 2	Nominal	98.2	100.2	106.5	99.2	102.6	99.1	101.2	18.8	24.6	30.3	11.0	19.6
	Offline	95.2	98.6	106.3	98.6	101.0	89.5	92.6	18.0	24.5	30.6	8.7	19.9
	Online	98.9	100.9	104.2	99.8	106.1	99.0	101.3	15.7	23.5	29.3	10.8	22.1
Patient 3	Nominal	99.9	101.5	107.4	98.5	100.3	99.1	100.9	28.6	19.2	37.1	12.4	30.6
	Offline	98.2	100.1	109.2	93.8	97.6	99.1	101.0	29.7	20.2	41.6	14.8	29.2
	Online	98.8	100.7	104.4	97.6	99.6	99.0	100.9	22.0	15.7	34.6	13.2	38.0
Patient 4	Nominal	98.2	100.2	106.4	98.8	100.5	98.4	100.4	21.2	19.8	2.8	0.37	15.7
	Offline	97.2	99.7	105.9	98.8	101.0	96.4	98.8	21.7	20.3	5.7	0.28	12.1
	Online	97.9	99.9	104.7	98.9	100.9	97.6	99.6	19.6	17.4	2.9	0.11	13.5
Patient 5	Nominal	99.0	100.6	106.4	–	–	99.2	101.6	34.9	24.9	20.9	3.2	7.0
	Offline	97.5	99.7	106.9	–	–	98.0	99.9	36.2	25.8	22.1	4.3	6.9
	Online	97.6	99.8	103.6	–	–	99.3	102.2	36.8	27.0	22.2	6.6	7.7
Patient 6	Nominal	98.4	100.3	106.2	98.7	100.3	99.5	101.5	25.4	41.8	47.0	1.0	4.0
	Offline	89.2	97.6	107.1	90.2	96.8	98.4	100.5	28.0	40.8	45.2	0.9	3.5
	Online	96.6	99.7	103.7	96.5	99.4	91.7	71.9	26.2	39.3	45.6	0.7	3.6
Patient 7	Nominal	99.0	100.5	108.1	99.7	102.3	99.0	100.5	12.9	32.0	26.0	15.1	15.9
	Offline	97.2	99.5	107.0	99.6	102.6	98.3	100.1	12.7	32.0	28.7	14.0	16.0
	Online	98.6	100.6	104.5	99.5	102.2	98.5	100.5	14.0	34.1	26.1	15.3	14.7
Patient 8	Nominal	88.4	93.9	106.1	–	–	–	–	7.2	7.2	53.7	18.8	26.6
	Offline	83.9	91.0	106.0	–	–	–	–	6.8	7.9	52.3	11.9	23.4
	Online	88.5	95.6	103.1	–	–	–	–	6.5	7.4	52.3	10.2	22.2
Patient 9	Nominal	98.3	100.3	106.4	–	–	98.2	100.4	25.5	25.4	4.9	12.6	17.0
	Offline	84.3	96.5	105.8	–	–	95.1	98.4	25.9	27.8	6.2	10.3	27.2
	Online	96.2	99.0	105.5	–	–	95.9	98.1	26.1	26.7	4.0	7.2	8.8
Patient 10	Nominal	99.7	101.0	105.9	–	–	99.6	100.6	24.3	25.1	33.1	6.2	3.0
	Offline	97.9	100.0	106.0	–	–	92.5	94.2	25.5	26.0	35.1	6.8	5.0
	Online	98.5	100.4	105.1	–	–	93.7	91.8	25.2	25.2	34.0	8.2	4.9

Abbreviations: AV_100_, the CTV percentage volume that received at least 100% of the prescription dose; *D*_98_, denotes the minimal dose received by at least 98% CTV volume, present in percentage of prescription dose; *D*_max_, the maximum dose received by CTV‐High, present in percentage of prescription dose; *D*_mean_, the mean dose of OAR; D_0.03cc_, the minimal dose received by the highest irradiated 0.03 cm^3^ of OAR.

**TABLE 4:** Quality scores calculated for the nominal plan, the cumulative dose of the offline APT course, and the cumulative dose of the simulated online APT course for the 10 patients.

		CTV‐High		CTV‐Mid	CTV‐Low	Right parotid	Left parotid	Oral cavity	Brainstem	Spinal cord	
		Score_V_100_	Score_D_max_	Score_V_100_	Score_V_100_	Score_D_mean_	Score_D_mean_	Score_D_mean_	Score_D_0.03cc_	Score_D_0.03cc_	Total score
Patient 1	Nominal	−7.7	5.2	2.2	6.1	7.0	7.0	7.0	7.0	7.0	40.8
	Offline	−19.5	3.7	−0.3	4.8	7.0	7.0	7.0	7.0	7.0	23.7
	Online	−18.0	6.3	2.2	6.2	7.0	7.0	7.0	7.0	7.0	31.7
Patient 2	Nominal	5.2	5.2	6.2	6.1	7.0	6.4	7.0	7.0	7.0	57.1
	Offline	0.3	5.3	5.6	−9.2	7.0	6.5	7.0	7.0	7.0	36.5
	Online	5.9	6.1	6.8	6.0	7.0	7.0	7.0	7.0	7.0	59.8
Patient 3	Nominal	6.9	4.3	5.5	6.1	1.8	7.0	2.4	7.0	7.0	48.0
	Offline	5.2	1.3	−2.0	6.1	0.6	7.0	−3.6	7.0	7.0	28.6
	Online	5.8	6.0	4.3	6.0	7.0	7.0	5.4	7.0	7.0	55.5
Patient 4	Nominal	5.2	5.2	5.8	5.4	7.0	7.0	7.0	7.0	7.0	56.6
	Offline	3.7	5.4	5.8	2.3	7.0	7.0	7.0	7.0	7.0	52.2
	Online	4.8	5.9	5.9	4.3	7.0	7.0	7.0	7.0	7.0	55.9
Patient 5	Nominal	6.0	5.2	–	6.2	−6.1	6.1	7.0	7.0	7.0	38.4
	Offline	4.2	5.0	–	5.0	−7.7	5.2	7.0	7.0	7.0	32.7
	Online	4.3	6.4	–	6.3	−8.5	3.8	7.0	7.0	7.0	33.3
Patient 6	Nominal	5.4	5.3	5.7	6.5	5.6	−14.8	−10.0	7.0	7.0	17.7
	Offline	‐9.7	4.8	−8.0	5.4	2.5	−13.5	−7.8	7.0	7.0	−12.3
	Online	2.7	6.3	2.5	−5.5	4.8	−11.6	‐8.3	7.0	7.0	4.9
Patient 7	Nominal	6.0	3.2	6.7	6.0	7.0	−2.5	7.0	7.0	7.0	47.4
	Offline	3.7	5.0	6.6	5.3	7.0	‐2.5	7.0	7.0	7.0	46.1
	Online	5.6	6.0	6.5	5.5	7.0	‐5.1	7.0	7.0	7.0	46.5
Patient 8	Nominal	‐11.0	5.4	–	–	7.0	7.0	−18.4	7.0	7.0	4.0
	Offline	−18.5	5.4	–	–	7.0	7.0	−16.6	7.0	7.0	−1.7
	Online	−10.8	6.6	–	–	7.0	7.0	−16.6	7.0	7.0	7.2
Patient 9	Nominal	5.3	5.2	–	5.2	5.5	5.6	7.0	7.0	7.0	47.8
	Offline	−17.8	5.5	–	0.2	5.1	2.8	7.0	7.0	7.0	16.8
	Online	2.0	5.6	–	1.5	4.9	4.1	7.0	7.0	7.0	39.1
Patient 10	Nominal	6.7	5.4	–	6.6	6.7	5.9	6.9	7.0	7.0	52.2
	Offline	4.8	5.4	–	‐4.2	5.5	5.0	4.9	7.0	7.0	35.4
	Online	5.5	5.8	–	‐2.2	5.8	5.8	6.0	7.0	7.0	40.7

*Note*: The scores were calculated for each planning criteria of our institutional planning guideline using the modified ProKnow scoring system.

**TABLE 5:** Score comparisons among the nominal plan, the cumulative dose of offline APT course and the cumulative dose of online APT course for the 10 patient cases.

		Patient 1	Patient 2	Patient 3	Patient 4	Patient 5	Patient 6	Patient 7	Patient 8	Patient 9	Patient 10	Average
Score	Nominal	40.8	57.1	48.0	56.6	38.4	17.7	47.4	4.0	47.8	52.2	41.0
	Offline	23.7	36.5	28.6	52.2	32.7	−12.3	46.1	−1.7	16.8	35.4	25.8
	Online	31.7	59.8	55.5	55.9	33.3	4.9	46.5	7.2	39.1	40.7	37.5
∆Score	Offline − Nominal	−17.1	−20.6	−19.4	−4.4	−5.7	−30.0	−1.3	−5.7	−31.0	−16.8	−15.2
	Online − Nominal	−9.1	2.7	7.5	−0.7	−5.1	−12.8	−0.9	3.2	−8.7	−11.5	−3.5
	Online − Offline	8.0	23.3	26.9	3.7	0.6	17.2	0.4	8.9	22.3	5.3	11.7

**FIGURE 3:**
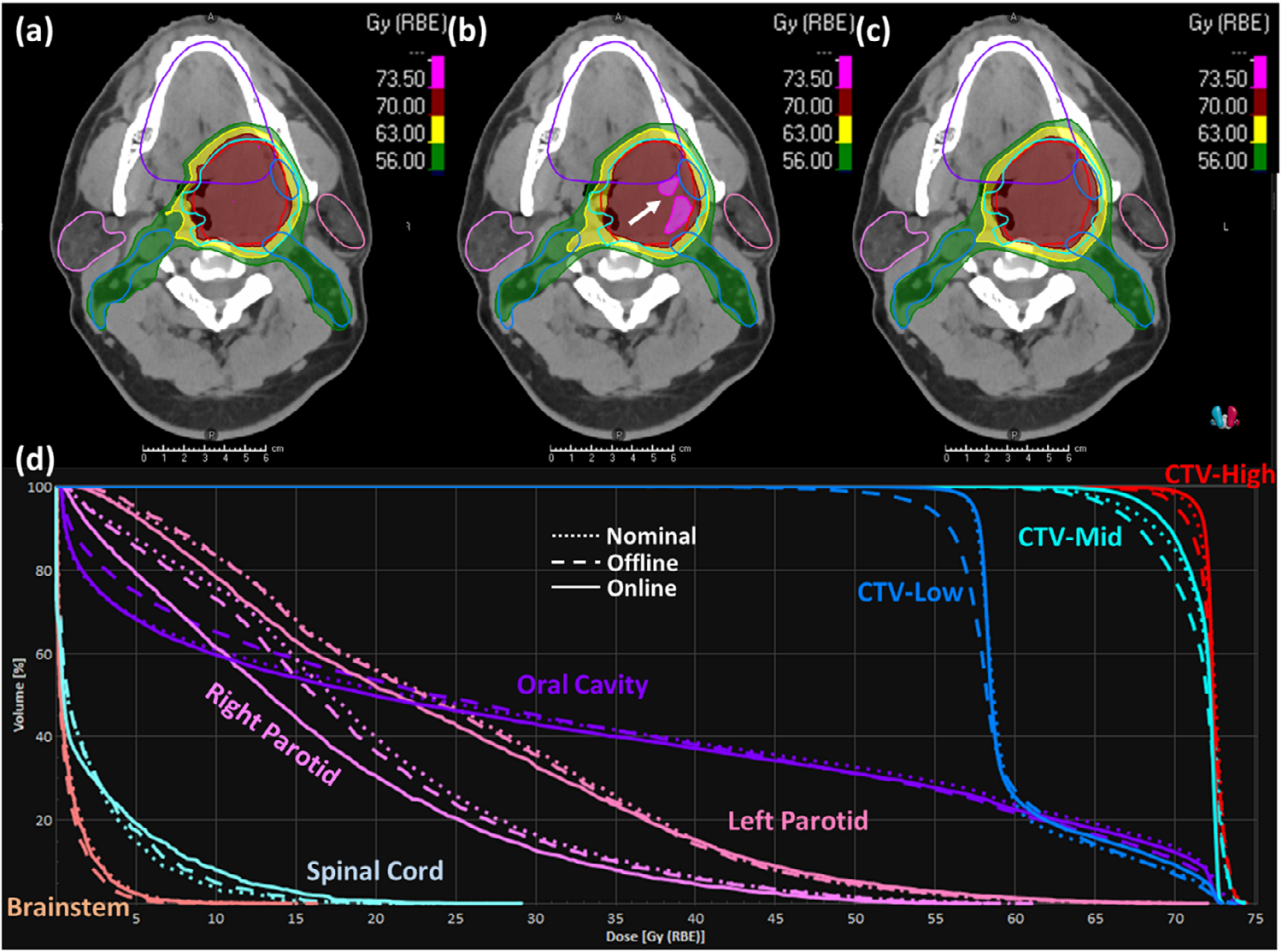
Dose comparison for patient 2 among the originally designed dose of the nominal plan, the cumulative dose of the actual offline APT treatment course, and the cumulative dose of our simulated online APT course. The top row displays the dose distributions in color wash for the nominal plan (a), the offline APT course (b), and the online APT course (c), overlaid with transverse CT images and the contours of CTVs and involved OARs shown in lines. The bottom row (d) displays the Dose‐volume histograms (DVHs) of CTVs and OARs, where the dot, dashed and solid lines represent the nominal plan, the offline course and the online course, respectively. The white arrow indicates the hot spot location.

**FIGURE 4:**
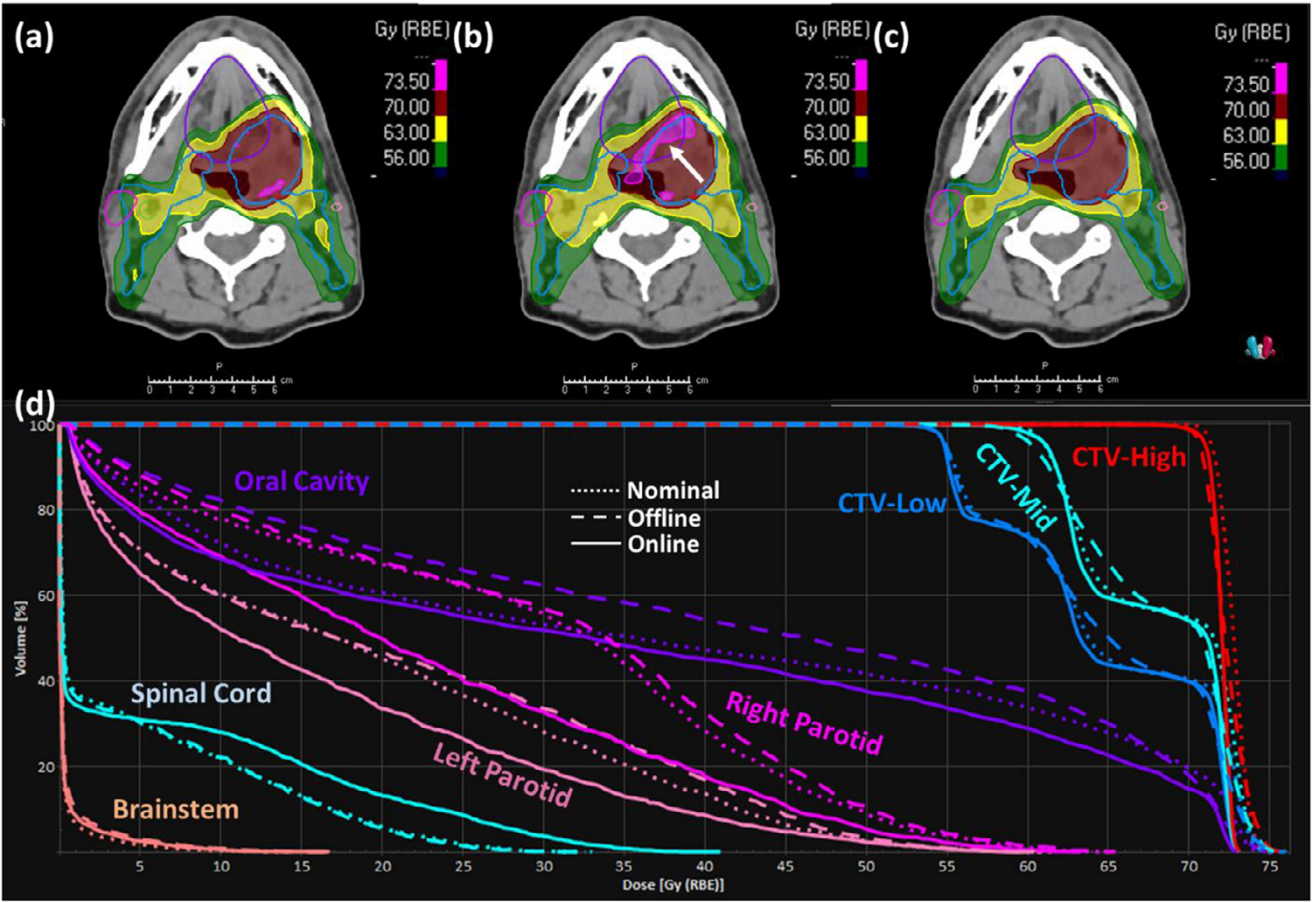
Dose comparison for patient 3 among the originally designed dose of the nominal plan, the cumulative dose of the actual offline APT treatment course, and the cumulative dose of our simulated online APT course. The top row displays the dose distributions in color wash for the nominal plan (a), the offline APT course (b), and the online APT course (c), overlaid with transverse CT images and the contours of CTVs and involved OARs shown in lines. The bottom row (d) displays the Dose‐volume histograms (DVHs) of CTVs and OARs, where the dot, dashed and solid lines represent the nominal plan, the offline course and the online course, respectively. The white arrow indicates the hot spot location.

**FIGURE 5:**
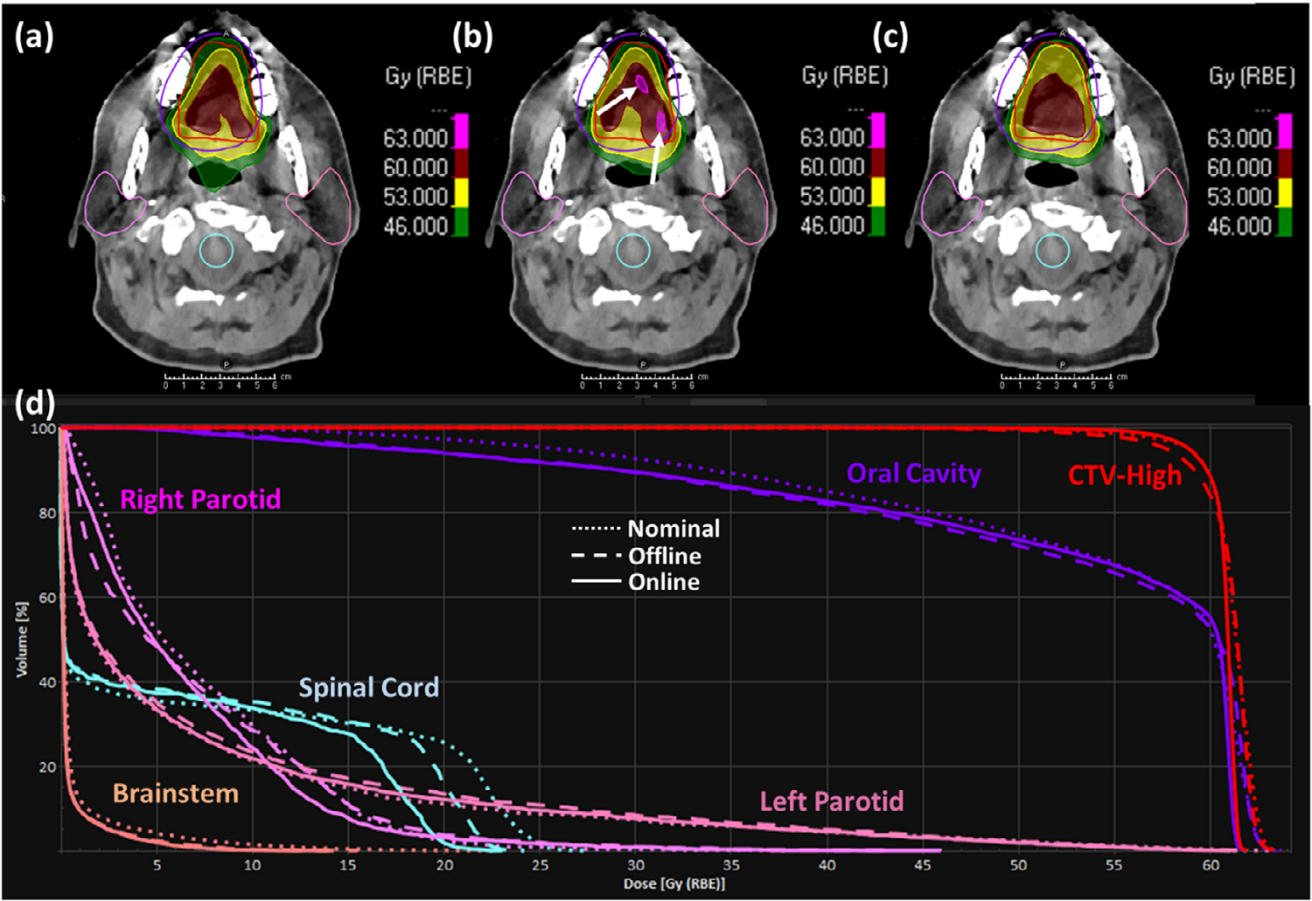
Dose comparison for patient 8 among the originally designed dose of the nominal plan, the cumulative dose of the actual offline APT treatment course, and the cumulative dose of our simulated online APT course. The top row displays the dose distributions in color wash for the nominal plan (a), the offline APT course (b), and the online APT course (c), overlaid with transverse CT images and the contours of CTVs and involved OARs shown in lines. The bottom row (d) displays the Dose‐volume histograms (DVHs) of CTVs and OARs, where the dot, dashed and solid lines represent the nominal plan, the offline course and the online course, respectively. The white arrows indicate the hot spot location.

It can also be observed from Table 5 that among the 10 patient cases, three cases (i.e., patient 2, 3, and 8) have a score of the simulated online APT course that not only surpasses that of the offline APT course substantially but also exceed the score of the nominal plan. These score comparison results are consistent with the dose volume endpoints presented in Table 3, as well as the DVHs of the three patients illustrated in Figures 3, 4, 5. Specifically, the offline APT course for patient 2 exhibits significantly lower CTV coverage for both CTV‐High and CTV‐Low compared to the nominal plan, with 95.2% and 89.5% versus 98.2% and 99.1%, respectively, and slightly reduced coverage for CTV‐Mid at 98.6% versus 99.2%. This results in a substantial decrease of the quality score from 57.1 to 36.5. In contrast, our simulated online APT course not only maintained the good coverage for all three CTVs, but also demonstrated small improvements compared to the nominal plan, resulting in a higher score of 59.8. Specifically, the coverages of CTV‐High and CTV‐Mid increased from 98.2% and 99.2% to 98.8% and 99.8%, respectively, with the hotspot of CTV‐High being improved from 106.5% to 104.2%. Meanwhile, the mean dose of left parotid was further reduced from 24.6 Gy‐RBE, which initially fell within but was close to our tolerance of 26 Gy‐RBE, to 23.5 Gy‐RBE. For patient 3, the nominal plan obtained the good CTV coverage at the cost of slightly sacrificing the sparing of the right parotid and oral cavity. The dose volume endpoints in Table 3 demonstrate that the offline replanning did not sufficiently address the patient's anatomical changes that occurred during the treatment course, yielding a worse coverage of CTV‐High (98.2% vs. 99.9%) and CTV‐Mid (93.8% vs. 98.5%), a higher hotspot inside CTV‐High (109.2% vs. 107.4%) that exceeds our clinical tolerance of 107%, and an even higher mean dose of right parotid (29.5 vs. 28.6 Gy‐RBE), and oral cavity (41.9 vs. 37.1 Gy‐RBE). In contrast, our online APT course not only maintained the good coverage of CTV‐High and CTV‐Mid at 98.8% and 97.6% respectively, but also effectively improved the endpoints that exceeded our clinical tolerances in the nominal plan to be within tolerances. Specifically, it reduced the hot spot of CTV‐High from 107.4% to 104.4%, the mean dose of right parotid from 28.6 to 22.2 Gy‐RBE, and the mean dose of oral cavity from 37.1 to 34.6 Gy‐RBE. Despite a significant increase in the spinal cord *D*_0.03cc_ dose from 30.8 to 38.1 Gy‐RBE, it remains well within our clinical tolerance of 54 Gy‐RBE. The total score achieved by our online APT course for patient 3 is 55.5, compared to 48.0 obtained by the nominal plan and 28.6 obtained by the offline APT course. As indicated by the notably low score of the nominal plan (total score of 4.0), the anatomy of patient 8 poses significant challenges for treatment planning and is manifested in the nominal plan with 88.4% coverage of CTV‐High and a mean dose of 53.7 Gy‐RBE for the oral cavity, both of which fell short of our clinical goals of achieving ≥98% coverage and a mean dose ≤35 Gy‐RBE. In this particularly challenging case, the offline APT course had difficulty in addressing the patient's anatomical changes, resulting in a significantly inferior CTV coverage of 83.9% and a total score of −1.7. In contrast, our simulated APT course surpasses the nominal plan with slightly improved CTV coverage (88.5% vs. 88.4%), and much lower hot spot (103.1% vs. 106.1%), and a reduced mean dose for the oral cavity (52.3 Gy‐RBE vs. 53.7 Gy‐RBE), which contributed to a higher total score of 7.2.

Except for these three cases, the score of our online APT course is lower than that of the nominal plan for all seven remaining cases. However, in comparison to the offline APT course, our simulated APT course exhibits a substantial score improvement in two cases out of the seven (i.e., an improvement of 22.3 for patient 9 and 17.2 for patient 6), a moderate improvement in three cases (i.e., an improvement of 8.0 for patient 1, 5.3 for patient 10, and 3.7 for patient 4), and a small improvement in two cases (i.e., an improvement of 0.6 for patient 5 and 0.4 for patient 7). As previously mentioned, our simulation study employed a template‐based replanning strategy for rapid online adaptation, omitting manual fine‐tuning of the priorities among multiple planning objectives, which is a step typically conducted for nominal treatment planning and offline replanning. While this approach reduces patient waiting time for online APT, it may constrain the extent of improvement achievable through online adaptation. To illustrate this effect, we selected patient 5, whose online APT course that was simulated without manual fine‐tuning resulted in a slightly higher score than the offline APT course, and performed one to two rounds of manual revision of priorities after the template‐based replanning. The dose volume endpoints and the resulting quality score for the fine‐tuned online APT course are presented in Tables 6 and 7, respectively. This additional manual tuning further improved the CTV‐High coverage achieved by online APT from 97.6% to 98.5% and reduced the mean doses of the right parotid and left parotid from 36.8 and 27.0 Gy‐RBE to 32.6 and 25.7 Gy‐RBE, respectively.

**TABLE 6:** Dose volume endpoints of CTVs and OARs for patient 5, obtained by the cumulative dose of the simulated online APT course without manual fine tuning and the simulated online APT course with further manual fine tuning (denoted as Online‐f), respectively.

	CTV‐High			CTV‐Mid		CTV‐Low		Right Parotid	Left Parotid	Oral Cavity	Brainstem	Spinal Cord
	*V*_100_ (%)	*D*_98_ (%)	*D*_max_ (%)	*V*_100_ (%)	*D*_98_ (%)	*V*_100_ (%)	*D*_98_ (%)	*D*_mean_ (Gy‐RBE)	*D*_mean_ (Gy‐RBE)	*D*_mean_ (Gy‐RBE)	*D*_0.03cc_ (Gy‐RBE)	*D*_0.03cc_ (Gy‐RBE)
Nominal	99.0	100.6	106.4	–	–	99.2	101.6	34.9	24.9	20.9	3.2	7.0
Offline	97.5	99.7	106.9	–	–	98.0	99.9	36.2	25.8	22.1	4.3	6.9
Online	97.6	99.8	103.6	–	–	99.3	102.2	36.8	27.0	22.2	6.6	7.7
Online‐f	98.5	100.5	104.6	–	–	99.5	102.9	32.6	25.7	22.2	6.3	7.8

*Note*: The endpoints obtained by the nominal plan and by the cumulative dose of the offline APT course are also represent here for comparison purpose.

Abbreviations: *D*_98_, the minimal dose received by at least 98% CTV volume, present in percentage of prescription dose; *D*_max_, the maximum dose received by CTV‐High, present in percentage of prescription dose; *D*_mean_, the mean dose of an OAR; *D*_0.03cc_, the minimal dose received by the highest irradiated 0.03 cm^3^ of an OAR; *V*_100_, the CTV percentage volume that received at least 100% of the prescription dose.

**TABLE 7:** Quality scores calculated for the cumulative dose of the online APT course without manual fine tuning and the online APT course with further manual fine tuning (denoted as Online‐f) for patient 5.

	CTV‐High		CTV‐Mid	CTV‐Low	Right parotid	Left parotid	Oral cavity	Brainstem	Spinal cord	Total score
	Score_V_100_	Score_D_max_	Score_V_100_	Score_V_100_	Score_D_mean_	Score_D_mean_	Score_D_mean_	Score_D_0.03cc_	Score_D_0.03cc_	
Nominal	6.0	5.2	–	6.2	−6.1	6.1	7.0	7.0	7.0	38.4
Offline	4.2	5.0	–	5.0	−7.7	5.2	7.0	7.0	7.0	32.7
Online	4.3	6.4	–	6.3	−8.5	3.8	7.0	7.0	7.0	33.3
Online‐f	5.5	6.0	–	6.5	−3.3	5.3	7.0	7.0	7.0	41.0

*Note*: The scores for the nominal plan and the cumulative dose of the offline APT course are also presented for comparison. The scores were calculated for each planning criteria of our institutional planning guideline using the modified ProKnow scoring system.

## 4 DISCUSSIONS

One challenge that we encountered in this retrospective study was the poor soft tissue contrast and large HU uncertainties of the CBCT images acquired by the on‐board CBCT imaging system of existing proton Linac systems, which prevented the direct use of the CBCT images for daily dose evaluation and online plan adaptation. To address this issue, we employed our previously developed CBCT correction framework,^15^ by first deforming the planning CT images to the daily CBCT images using the deformable registration algorithm provided in RayStation and then transferring the air cavities from the CBCT images to the deformed planning CT images. This strategy aimed to utilize the accurate HU values from the planning CT images while persevering the patient's actual treatment anatomy captured in the CBCT images. It is noteworthy that this strategy relies on the accuracy of the deformable image registration. On one hand, there are a few studies^30^, ^31^ from other groups that have also validated the dosimetry accuracy of the DIR‐based method for daily dose evaluation and online adaptation for proton therapy. On the other hand, our specific focus in this study was to compare the effectiveness of online APT and offline APT in the presence of substantial anatomical changes for HN cancer patients. Despite potential imperfections in the deformable registration process, the same corrected CBCT images were used to accumulate the actual dose for both online and offline APT courses to maintain the consistency, which allowed us to draw reasonable conclusions from our comparison results. Meanwhile, many groups have been devoting efforts to enhancing the image quality and HU accuracy.^32^ Advanced deep learning technologies have been employed to generate synthetic CT images from CBCT images,^33^, ^34^ presenting an alternative solution for CBCT‐guided online APT. Furthermore, a new on‐board CBCT imaging system named HyperSight (Varian Co., Palo Alto, CA, USA) has been developed for the newer model of the CBCT‐based online adaptive photon therapy system Ethos very recently, which offers CBCT images of significantly improved quality. The potential translation of this new CBCT imaging technology to proton systems in the future holds promise for CBCT‐guided online APT.

Manual contouring was deemed impractical for online APT due to its labor‐intensive nature. In this study, we propagated the OAR contours from the planning CT images onto the corrected CBCT images through either rigid copying or deformable mapping of contours specific to each OAR, depending on its characteristics. This approach is similar to the OAR contour propagation from planning CT images to QACT images in the current clinical practice of offline APT, and relies on the accuracy of image registration. Considering recent advancements in on‐board CBCT imaging and deep learning methods for CBCT‐based synthetic imaging, organ segmentation emerges as a viable alternative for achieving automated OAR contouring in CBCT‐based online APT. Given logistical constraints and cost considerations, acquiring daily MRI scans throughout the 4−6 week treatment course to assess tumor regression status and update CTV volumes was deemed impractical. Instead, deformable image registration was performed to propagate the original CTV contours manually delineated by physicians on the planning CT images either onto the corrected CBCT images for our simulated online APT or onto the QACT images in our current clinical practice for offline APT. The assumption here is that the features and relationships between the CTVs and nearby anatomical structures present on the planning CT images are preserved in the daily CBCT images that reflect the patient's actual treatment anatomy. The same strategy of CTV contour propagation was employed by Ethos for CBCT‐based online adaptive photon therapy.^35^ It is acknowledged, however, that this CTV contour propagation strategy may not seamlessly accommodate tumor volume changes into plan adaptation for patients experiencing rapid tumor changes. In such cases, more frequent acquisition of MRI scans, possibly on a weekly basis, may be necessary during the treatment course to aid in CTV delineation on the corrected CBCT images for both online and offline adaptation. Importantly, as our study maintains consistency by applying the same CTV propagation strategy for both online and offline APT courses and using the identical CTV contours on the daily CBCT images that have been reviewed by physicians for dose accumulation in both courses, our comparison results between the two APT courses in this specific study are fair, allowing for reasonable conclusions to be drawn.

In our retrospective study, due to the unavailability of online APT technology, we simulated the online replanning process by employing a template‐based automated replanning strategy that was developed by Ethos for CBCT‐based online replanning for photon therapy.^23^, ^24^ It is crucial to note that the observed improvements achieved by our simulated online APT course, compared to the actual offline APT course, result from a combination of online adaptation based on patient's actual treatment anatomy and our choice of using a smaller setup margin (e.g., 1.5 mm vs. 3 mm). The smaller setup margin is only applicable in the context of online adaptation to account for potential intra‐fractional motion and spot positioning uncertainty, because the patient's actual anatomy in treatment position, that is, captured in the CBCT image for each fraction is used to conduct the online dose evaluation and online adaptation, if necessary, prior to treatment delivery of each fraction. Therefore, online adaptation offers the advantage of allowing a smaller setup margin or uncertainty to reduce the doses delivered to nearby OARs. Our simulation results have demonstrated that even with the much smaller setup margin of 1.5 mm, online adaptation can still maintain good CTV dose coverage. Furthermore, our choice of a 1.5 mm setup margin for online adaptation aligns with a very recent dosimetry study on HN proton therapy by Bobić et al.^36^ in which they used a 4 mm setup margin for offline replanning and a 1 mm setup margin for online replanning. Using a smaller setup margin for online adaptation was also suggested in multiple other studies on adaptive radiotherapy^37^, ^38^ A recent review paper by Huiskes et al. on adaptive proton therapy in HN cancer noted that the majority of online adaption studies did not use any setup margin.^39^ We would like to mention that our determination of the 1.5 mm margin size was based on a study on intra‐fractional motion of HN cancer patients^25^ and a study on spot positioning uncertainty.^26^ The optimal margin size for each individual patient needs further investigation in the future with the technology of online APT being in place. Unlike online adaptation, offline replanning is performed between treatment fractions by acquiring a repeated CT scan and then repeating the entire planning process to create a new plan for the remaining fractions based on this new CT scan. Due to the substantially increased clinical workload associated with this offline replanning process, a relatively large setup uncertainty is often employed in the original treatment planning and offline replanning in order to reduce the instances of needed offline replanning. However, this comes at the cost of delivering higher doses to neighboring normal tissues and OARs. Meanwhile, using a small setup margin or uncertainty in offline adaptation is not clinically feasible and would compromise target coverage.

Rapid plan adaptation is imperative to meet the stringent time requirements for online APT, contributing to the challenges in implementing online APT technology, which is not yet available in proton clinics. In this simulation study, the execution of steps 1−7 of the proposed online adaptation workflow (Figure 1) took about 2 min. For step 8 of the online replanning, we employed a template‐based automatic replanning strategy, that is, utilizing the same priorities among multiple planning objectives that were specified in the original treatment planning for robust plan optimization without additional manual fine‐tuning. With a maximum of 150 iterations set for the robust plan optimization that involves three scenarios, step 8 took approximately 10−15 min for each patient. Despite the simplicity of this template‐based replanning, the quality of our simulated online APT courses of three patients not only significantly surpassed the offline APT course but also exceeded the nominal plan. Among the seven remaining patients, our simulated APT course exhibited substantial score improvement in two cases, moderate improvement in three cases, and small improvement in two cases (i.e., patient 5 and 7). For the sake of efficiency, the template‐based replanning strategy omits the manual fine‐tuning of priorities, which, however, is a step typically conducted for original nominal plans and offline replanning. This may limit the extent of quality improvement achievable through online adaptation, as indicated by the results in Tables 6 and 7 that compare online adaptation with and without additional fine‐tuning for patient 5. Considering the time taken by contour and plan review, as well as contour modification and plan fine‐tuning if needed, we estimate that the entire plan adaptation process for proton therapy for HN cancer would fall within 30−45 min. This duration is comparable to other online ART modalities (such as Ethos and MR‐Linac). On the other hand, it is important to note that the proton beams are often shared by multiple treatment rooms in most proton centers, and patients must wait for treatment delivery while the proton beam is used in other rooms. The online adaptation process in one room will not prevent the proton beam from being delivered to other rooms. Methods to improve proton patient scheduling and “on‐the‐fly” decision‐making on patient call‐back time may be needed to seamlessly incorporate online APT into routine clinical workflows and utilize proton beams more efficiently. Furthermore, the recent successes in deep reinforcement learning‐based automatic treatment planning across various treatment modalities and cancer types, including IMRT for prostate cancer^27^, ^28^, ^29^ and pancreas cancer,^40^ brachytherapy for cervical cancer,^41^, ^42^ and Gamma Knife radiosurgery for vestibular schwannoma^43^ have demonstrated an intelligence in priority tuning comparable to experienced human planners. This technology holds the potential to further extend potential benefits of online adaptation without the need for manual fine‐tuning.

One limitation of this study is the small patient cohort consisting of only 10 cases, which is attributed to the substantial workload associated with conducting this retrospective simulation study, such as deformable image registration of the planning CT images to the daily CBCT images and contour propagation, review, and modification at every treatment fraction, dose recalculation for the in‐use plan of the online APT course and the in‐use plan of the offline APT course at very fraction, dose evaluation at every fraction of the online APT course, replanning whenever online adaptation is triggered, deformable registration of the CBCT images of every fraction to the planning CT images for dose accumulation over every fraction for both online and offline APT courses. However, the selection of these 10 cases aimed to encompass all the major factors leading to offline replanning, such as CTV coverage loss, increased hot spots in high‐risk CTV, deteriorated OAR sparing, and obvious anatomical changes noted from CBCT images (as outlined in Table 1). Among the 10 patient cases, there are several cases which encountered various planning challenges during the nominal treatment planning. For instance, the CTVs of patient 1 are in close proximity to the brainstem, which posed planning challenges related to the sparing of brainstem. As presented in Table 3, the brainstem *D*_0.03cc_ dose obtained by the nominal plan for patient 1 was 62.9 Gy, which fell just within our clinical tolerance of 63 Gy. Patient 3 encountered planning challenges related to sparing both right parotid and oral cavity, as indicated by the dose volume endpoints of the nominal plan presented in Table 3. Similarly, patient 6 had planning challenges related to sparing both left parotid and oral cavity. Patients 5 and 7 had challenges related to the sparing of right parotid and left parotid, respectively. Patient 8 encountered planning challenges related to CTV dose coverage and sparing of the oral cavity. Additionally, as mentioned before, the score values of the nominal plans of the 10 patients also serve as indicators of the planning difficulty level for each patient to some extent. The wide score range presented in Table 5, ranging from 4.0 (obtained for patient 8) and 57.1 (obtained for patient 2), illustrates a substantial variety in patient anatomy in terms of planning difficulty level.

The results of our retrospective study underscore the variability in the potential benefits of online adaptation among patients, influenced by factors such as the extent of anatomical changes and the proximity of critical organs. Given the additional clinical resources, including time and personnel, required for online APT, an optimal approach would involve pre‐treatment identification of patients likely to gain substantial benefits from online APT. This allows for a targeted application of online APT for those individuals, while others proceed with conventional non‐adaptive or offline adaptive proton therapy. In our ongoing efforts, we plan to expand our retrospective study to encompass a larger cohort of patient cases. This extended analysis aims to elucidate the characteristics of patients exhibiting significant benefits from online adaptation. By discerning patterns from a more extensive dataset, we seek to identify those individuals most likely to gain substantial benefit from online APT. Our future research endeavors also include an exploration of the potential impact of online APT on treatment outcomes. This involves assessment of Tumor Control Probability (TCP) and Normal Tissue Complication Probability (NTCP) based on the cumulative dose of the simulated online APT course. While various TCP and NTCP models exist,^44^, ^45^, ^46^, ^47^ the accurate determination of model parameters specific to each tumor type or normal tissue type is crucial and necessitates thorough investigation based on the available clinical dose–response data. A key focus of our forthcoming investigations is to identify optimal values for model parameters relevant to head and neck (HN) tumors considered in this simulation study, which is essential to ensure the accuracy and reliability of the estimated TCP and NTCP values.

## 5 CONCLUSIONS

To investigate the potential dosimetric benefit of online APT and compare it with the current offline APT practice, we conducted a retrospective study on 10 HN cancer patients who had at least one offline replanning throughout their actual IMPT treatment courses and simulated a CBCT‐guide online APT course for each patient using the daily CBCT images acquired from each treatment fraction of the actual offline APT course. The ProKnow scoring system was employed and adapted for our study to quantify the treatment quality of the simulated online APT course against our clinical planning goals and compare with that of the actual offline APT course and the originally designed dose of the nominal plan. The average score of the nominal plans across the 10 patient cases was 41.0, while the average score for the actual offline APT course was 25.8, and the average score for our simulated online APT course was 37.5. Relative to the offline APT course, the simulated online APT course improved the quality score for all 10 cases, with enhancements ranging from 0.4 to 26.9 and an average improvement of 11.7. Notably, the quality scores of our simulated online APT courses of three patients not only significantly surpassed the offline APT course but also exceeded the nominal plan. Among the remaining seven patients, relative to the offline APT course, the simulated APT course exhibited substantial score improvement in two cases, moderate improvement in three cases, and slight improvement in two cases. Considering the diverse patient anatomy and varying planning complexity levels across the 10 patient cases, we consider that our simulation results are representative of the broader patient population treated at our institution and have demonstrated that online APT can better address anatomical changes for HN cancer patients than the current offline replanning practice. The advanced artificial intelligence‐based automatic replanning technology^27^, ^28^, ^29^, ^40^, ^41^, ^42^, ^43^ presents a promising avenue for extending the potential benefits of online APT.

## AUTHOR CONTRIBUTIONS

Chih‐Wei Chang: method development, manuscript writing, data collection and processing. Duncan Bohannon: data collection and processing and patient screening. Zhen Tian: manuscript writing and method development. Yinan Wang: treatment planning and data collection. Mark W. Mcdonald: physician advice for treatment planning. David S. Yu: physician advice for treatment planning. Tian Liu: manuscript writing. Jun Zhou: method development and treatment planning. Xiaofeng Yang: supervising the project and research direction and manuscript writing.

## CONFLICT OF INTEREST STATEMENT

The authors declare no conflicts of interest.
